# Effectiveness of Virtual Surgical Planning and Three-Dimensional Anatomical Models in Radiological Reconstruction of Center of Rotation and Pelvic Brim in Patients with Anterior Column Defects Requiring Revision Hip Arthroplasty

**DOI:** 10.3390/diagnostics14222574

**Published:** 2024-11-15

**Authors:** Krzysztof Andrzejewski, Marcin Domzalski, Bozena Rokita, Jan Poszepczynski, Piotr Komorowski

**Affiliations:** 1 Veteran’s Memorial Hospital Medical, University of Lodz, Zeromskiego 113, 90-549 Lodz, Poland; kj.andrzejewski@outlook.com (K.A.); marcin.domzalski@umed.lodz.pl (M.D.); janek24061982@tlen.pl (J.P.); 2 Institute of Applied Radiation Chemistry, Faculty of Chemistry, Lodz University of Technology, Wroblewskiego 15, 93-590 Lodz, Poland; bozena.rokita@p.lodz.pl; 3 Division of Biophysics, Institute of Materials Science, Lodz University of Technology, Stefanowskiego 1/15, 90-924 Lodz, Poland

**Keywords:** revision hip arthroplasty, oversizing technique, dual-mobility cup, anterior column defect, pelvic brim discontinuity

## Abstract

**Background:** The aim of this study was to show that virtual surgical planning (VSP) and printed anatomical models support the reconstruction of the center of rotation (COR) and pelvic BRIM during revision hip surgery using a dual-mobility revision cup system in patients with anterior pelvic column damage and soft tissue envelope deficiency. **Methods:** Patients with anterior pelvic column damage and soft tissue envelope deficiency underwent revision hip arthroplasty. Virtual planning included assessment of bone segmentation, positioning of the cementless revision cup while maintaining the COR, and the assumed inclination and anteversion angles. **Results:** The diameter of the planned revision cups was 65.5 ± 2.1 mm, and the diameter of the revision cups used was 65.3 ± 2.1 mm. The difference in COR position in the horizontal axis was 7.8 ± 9.3 mm, in the vertical axis was 4.3 ± 5.9 mm, and in the axial plane was 1.6 ± 3.3 mm. The differences in inclination angle and in the anteversion angle were 12.4° and 8.7°, respectively. **Conclusions:** The use of VSP and 3D models supports the process of performing RHA surgery in patients with damage to the anterior pelvic column and soft tissue envelope deficiency.

## 1. Introduction

**Figure 1:**
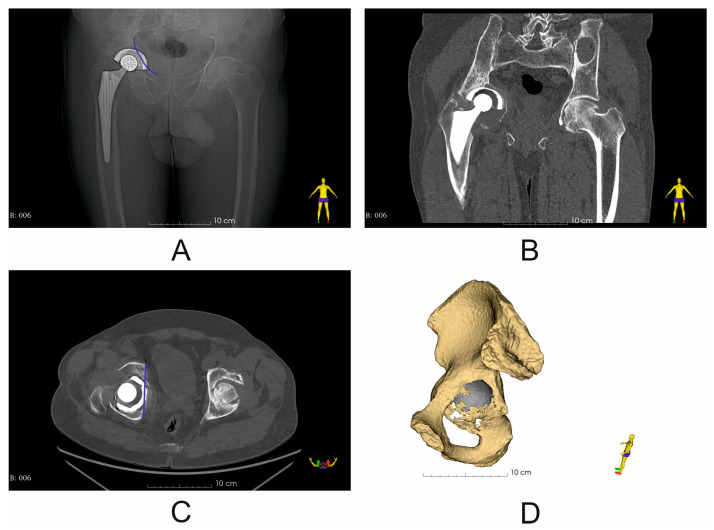
Representative imaging results (X-ray and CT scan) (Patient No. 6) with marked pelvic BRIM defect (blue line) (**A**,**C**); (**A**) X-ray image before revision surgery; (**B**) coronal plane image of CT scan before revision surgery; (**C**) axial plane image of CT scan before revision surgery; (**D**) segmented pelvic bone with visible structural discontinuity of the iliopectineal line (pelvic BRIM).

The 21st century has seen a huge increase in the number of primary total hip arthroplasty (THA) procedures performed, with data indicating that by 2030 the demand for THA will increase to 173% in the US market, with similar predictions for Australia and the UK [1]. As a consequence, there has been a significant increase in the number of revision total hip arthroplasty (RHA) procedures, with RHA in most cases affecting the acetabular component, which is characterized in radiological images by a violation of the iliopectineal line (BRIM) and ilioischial line; the lines represent the continuity of the anterior and posterior columns (Figure 1) [2]. An essential element of revision surgery is to fill the bone defects that have arisen following the removal of the loose cemented or uncemented primary implant with an appropriate material and to achieve primary stabilization of the implant. It is necessary to recreate the center of rotation (COR), the anatomical outline of the pelvic BRIM, and maintain the correct inclination and anteversion angles. This, combined with the undisturbed process of implant osseointegration, guarantees a good early and late postoperative result [3]. Currently available on the market, cementless revision cups and augment systems allow for complete filling of bone defects created after the primary implant placement, as classified by Ghanem and Adreas as type III [4] or more popular classifications among orthopedists, e.g., by Paprosky as type IIIA and IIIB [5] or the American Academy of Orthopaedic Surgeons (AOOS) classification as III [4]. Additionally, the development of the implant–bone contact surface by increasing its surface roughness and the use of titanium alloys (Ti6Al4V) or tantalum and multi-hole cups allow for primary stabilization during the procedure, while maintaining the correct angles of inclination and anteversion, as well as the reconstruction of the BRIM and COR of the pelvis [6]. The portfolio of orthopedic implant systems is complemented by custom-made systems, which constitute a separate direction in orthopedics [7]. It is generally believed that the most difficult cases involve patients who have not only up and/or out defects, but also significant defects of the anterior part of the pelvic column (BRIM pelvic defect) [3,6]. Despite the fact that this type of defect constitutes about 5% of defects, the correct surgical treatment is crucial to achieve correct biomechanics of the pelvic bones [8]. According to the results of the study by Thossart Harnroongroj et al. [9], the anterior column provides 2.75 times greater strength to the pelvic ring than the posterior column. This means that the anterior column has more pronounced biomechanical importance for the stability of the pelvic ring than the posterior column, even though its contour is smaller and thinner [9]. The surgical technique used in such cases involves filling the defect as thoroughly as possible with a cementless large-diameter acetabular implant (oversizing technique), stabilized with screws in the pelvic bone roof and in the posterior quadrant of the acetabulum, e.g., using allogeneic grafts. Moreover, the use of the dual-mobility (DM) system provides good clinical results in patients with soft tissue envelope deficiencies or polyethylene insert wear, in whom the acetabulum has not loosened [10].

The hypothesis of our technical note is that the use of virtual preoperative planning (VSP) and 3D anatomical models introduced into clinical practice will effectively support the planning and carrying out of revision surgeries in patients with anterior pelvic column defects and soft tissue deficiency. The aim of this study is to investigate whether this method allows preoperative selection of an appropriate large-diameter revision cup (oversizing technique) and correct reconstruction of the COR and BRIM of the pelvis, while maintaining appropriate inclination and anteversion angles in patients requiring RHA.

## 2. Materials and Methods

### 2.1. Clinical Information and Surgical Technique: Classification of the Acetabular Bone Defect

In the period from January 2022 to December 2023, a total of 8 patients (4 male and 4 female), aged 65 to 70 years (mean age 67 years), with anterior column damage and soft tissue envelope deficiency, who underwent single-stage revision hip arthroplasty (RHA), were included in this study. The study population was homogeneous. All patients had undergone primary unilateral THA with cemented or uncemented acetabular cups that had become aseptic loosened. All patients underwent imaging examinations, i.e., X-rays and CT scans, before and 7 days after the procedure. All patients had a damaged anterior pelvic column with lysis of the pubic bone and a damaged iliopectinal line, as visualized on X-ray and CT images, as wells as a positive Trendelenburg sign. Patients were qualified for the study according to Ghanem and Andreas classification type III without pelvic discontinuity [4]. The same implant system was used in all patients, i.e., the AESCULAP^®^ Plasmafit Revision^®^ dual-mobility system (Aesculap AG, Tuttlingen, Germany). The diameter of the acetabulum used was 62–66 mm (female) and 64–68 mm (male), and the diameter of the metal head was 28 mm (Table 1). In case of insufficient contact between the revision metal acetabulum cup and the pelvic bone, an allogeneic cancellous bone graft was used (Tissue Bank, Katowice, Poland). Revision cups were supported with two screws placed in the acetabular roof (Table 1). Revision surgery was performed using a posterolateral approach (Kocher–Langenbeck) with the patient lying in a lateral decubitus position. All patients were operated on by the same surgical team and orthopedic department. This study was approved by the Ethics Committee on Human Research of the Medical University of Lodz, 117 No. RNN/126/22/KE.

### 2.2. Virtual Surgical Planning (Pre- and Post-Operation Assessment): Assessment of the Quality of Bone Structure Segmentation and Pelvic BRIM Damage, Determination of the Diameter of the Revision Cup, Determination of the Inclination and Anteversion Angles and the Center of Rotation (COR), and Assessment of the Size of the Acetabular Revision Cup

**Figure 2:**
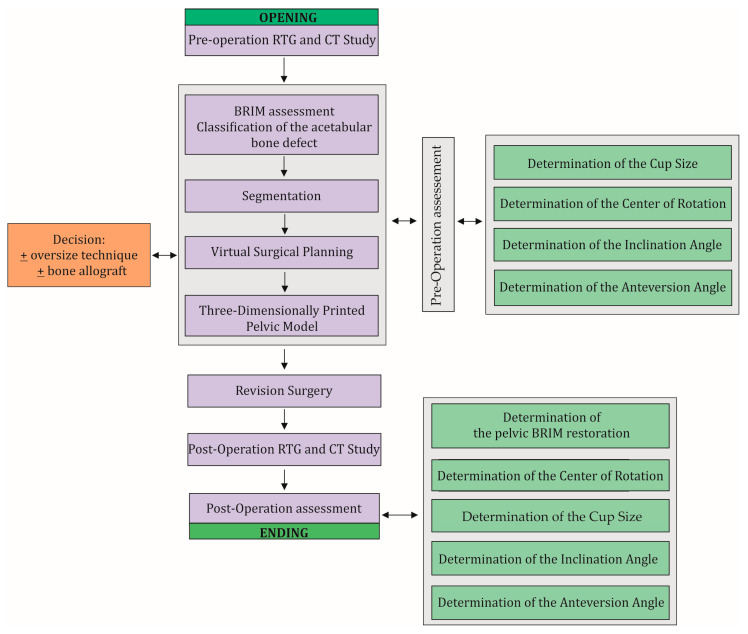
The workflow used in this study and clinical practice. The workflow consists of two main stages of the procedure, i.e., pre-operation evaluation and post-operation evaluation, which include the same steps that need to be performed. During the VSP, a decision is made on the surgical technique to be used and the possible use of a bone allograft. A necessary step in this procedure is to perform X-rays and CT scans before and after the revision surgery.

**Figure 3:**
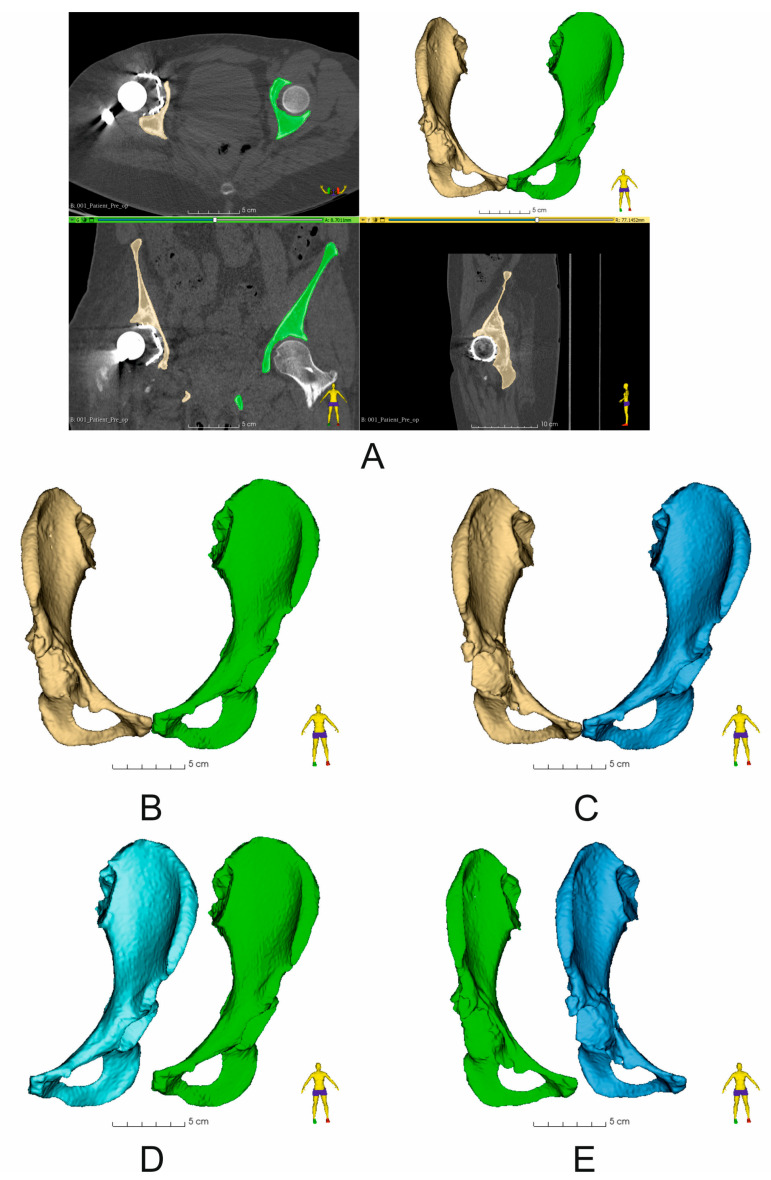
Assessment of bone structure segmentation and comparison of the volume of segmented bone structures within the operated pelvis (Patient No. 7): (**A**) Image of segmented pelvic bones (yellow—damaged side of the pelvis, green—undamaged side of the pelvis) obtained on the basis of CT image analysis. View in the following planes: axial, coronal, and sagittal; (**B**) comparison of the volume of undamaged (yellow) and damaged (green) pelvic bones before revision surgery; (**C**) comparison of the volume of undamaged (yellow) and damaged (blue) pelvic bones after revision surgery; (**D**) comparison of the volume of undamaged sides of the pelvic bone: blue—undamaged pelvic bone after revision surgery, green—undamaged pelvic bone before revision surgery; (**E**) comparison of the volume of damaged sides of the pelvic bone: blue color—damaged pelvic bone after revision surgery, green color—damaged pelvic bone before revision surgery. The numerical data are shown in Table 2.

**Figure 4:**
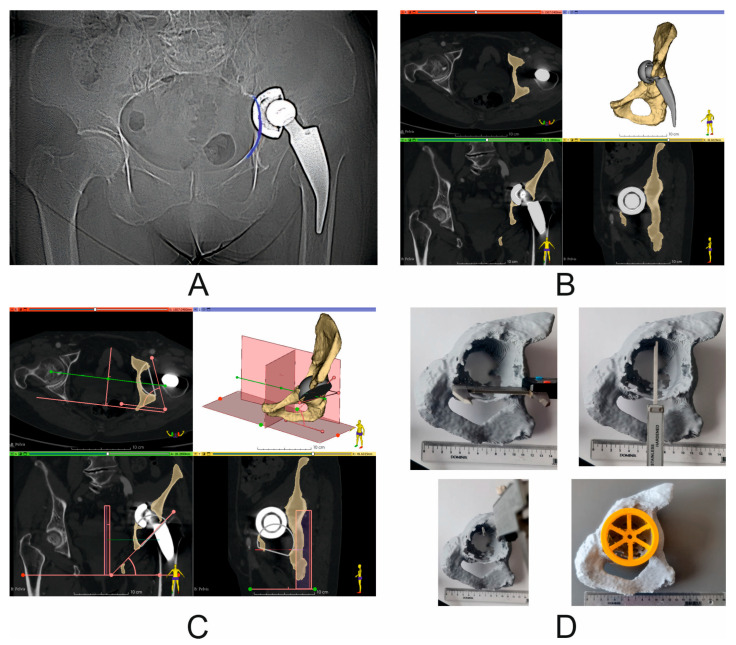
Virtual COR and pelvic BRIM reconstruction, based on the assumed inclination angle and anteversion angle according to the Lewinnek safe zone, and taking into account the smallest possible bone loss. (**A**) X-ray image (Patient No. 2)—blue color—marked discontinuity of pelvic BRIM; (**B**) preoperative CT scan (Patient No. 2). View in the following planes: axial, coronal, and sagittal; segmented bony structures of the pelvis; yellow color—damaged pelvis with bony defect of the anterior column and pubic bone and visible damage to pelvic BRIM; gray color—primary acetabulum and stem with head; (**C**) virtual surgical planning—virtual reconstruction of the COR and pelvic BRIM based on the assumed inclination angle and anteversion angle according to the Lewinnek safe zone; yellow color—simplified representation of a 68 mm diameter revision cup; (**D**) photo of a printed model of a damaged pelvis showing the way of measuring the distance between the most important support points for the revision cup. This is characteristic for preoperative planning measurement between the pubic bone and the ischium, and between the acetabular notch and the roof. For the oversizing technique, the quotient is close to 1 (i.e., the ratio of horizontal axis defect distance to vertical axis defect distance in the sagittal plane). Verification of the cup diameter selection using the printed cup gauge—diameter 68 mm (Patient No. 6).

**Figure 5:**
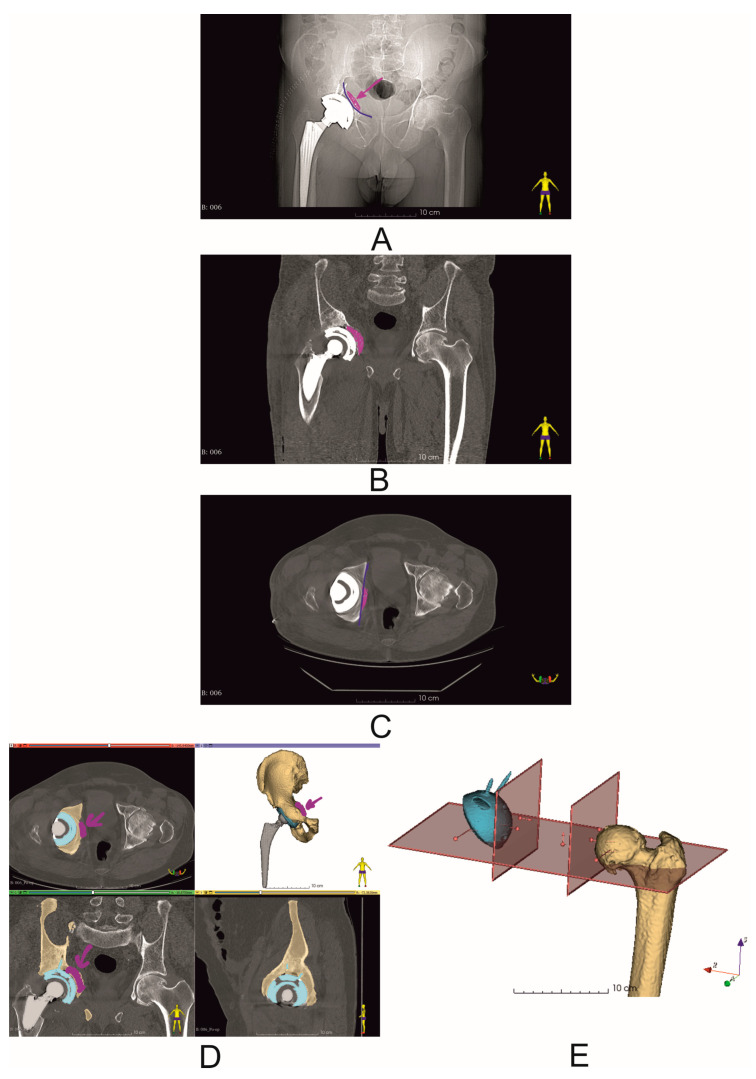
Postoperative evaluation of the position of the dual-mobility revision cup (Patient No. 6). Blue lines indicate the reconstructed pelvic BRIM. The purple arrow indicates the bone allograft used during the procedure. (**A**) X-ray image; (**B**,**C**) postoperative CT scans; (**D**) segmented bone structures of the operated cup, with the allograft position marked with an arrow (purple), blue color—dual-mobility revision cup with screws; (**E**) postoperative analysis of the COR position and inclination and anteversion angles.

Computed tomography (CT) scans were performed in all patients before and 7 days after surgery using one of the following scanners, Siemens Sensation Cardiac 64 (Siemens, Munich, Germany) or GE Medical Systems Optima CT540 (GE Healthcare, Chicago, IL, USA), according to the established CT study protocol [11] and the following workflow (Figure 2). Virtual surgical planning (pre- and post-operation assessment) was performed using 3D Slicer 5.6.2 software [12]. First, the disruption of the pelvic BRIM was determined, then the correctness of the segmentation process was checked by comparing the volume of the undamaged part of the pelvic bone before and after the surgery (based on the results of two CT scans before and after the procedure) (Figure 3). Then, the percentage of the anterior column defect on the operated side was determined in relation to the undamaged side, as well as the operated side before and after the surgery (Figure 3 and Table 2). The next step of the VSP process was the selection of the surgical technique, i.e., assessing the need for the oversize technique and the need for allograft application. Then, virtual preoperative planning was performed by determining the size (Table 1) and positioning of the cementless revision cup, keeping the implemented element position as close as possible to the specified COR, taking into account the BRIM reconstruction of the pelvis and the assumed inclination and anteversion angles within the Lewinnek safe zone, and considering the minimum bone loss caused by the use of cup reamers to prepare the revision cup site [11,13,14] (Figure 2 and Figure 4, and Table 3). A 3D pelvic model was then prepared and printed to perform physical measurements of bone loss (Figure 4). After completion of the VSP procedure, revision surgery (on the patient) was performed. The final step of the entire procedure was the postoperative assessment of the position of the dual-mobility revision cup using X-ray and CT, taking into account the parameters considered during the pre-planning, including the cup size (Figure 5, Table 2 and Table 3).

### 2.3. Three-Dimensional Printing of Pelvic Models

CT data were segmented and the resulting 3D models were exported as stl files and later imported into CURA 5.6.0 3D printing software (Ultimaker B.V, Geldermalsen, The Netherlands), which was used to configure the printing process, and the pelvis models were printed using the Ultimaker U2+ printer according to the parameters described in Table 4 [11].

### 2.4. Statistical Analysis

OriginPro 2023 software (OriginLab Corporation, Northampton, MA, USA) was used for statistical analysis. The results are presented as mean ± standard deviation (SD), and the Shapiro–Wilk test was used to test normality. Differences between pre- and postoperative results were evaluated by a paired *t* test or Wilcoxon test. Correlations between the variables were determined using the Pearson correlation coefficient. Differences between mean values were considered as statistically relevant, with a significance level of * *p* < 0.05.

## 3. Results

Based on the assessment of the correctness of the pelvic bone segmentation performed in the first stage of virtual planning, the differences in the volume between the undamaged side of the pelvic bone before and after the revision surgery were shown to be 0.01 ± 0.005% (statistically significant). On the other hand, the analysis of the percentage of the pelvic bone defect including the anterior column between the undamaged and the damaged pelvic bone before the revision surgery showed an average value of 25.2 ± 5.8% (statistically significant). The next analyzed parameter was the percentage of the anterior column bone defect between the undamaged and damaged pelvic bone; after the revision surgery, it amounted to 30.1 ± 6.7% (statistically significant). Finally, the percentage of anterior column bone loss between the damaged pelvic bones before and after the revision surgery was determined, and the value of this loss was 5.0 ± 2.0% (statistically significant) (Table 1). The range of revision cup sizes used was in the range from 62 to 68 mm (62–66 mm for females and 64–68 mm for males). The overall mean diameter of the planned revision cups (preoperative results) was 65.5 ± 2.1 mm, and the mean diameter of the revision cups used (postoperative results) was 65.3 ± 2.1 mm. Bone allografts were used in all cases (Table 1). The difference in the COR position for the operated side of the pelvis in the vertical axial plane (coronal plane) between the preoperative and postoperative results was 7.8 ± 9.3 mm (statistically significant), while the difference in COR position in the horizontal axial plane (coronal plane) for preoperative vs. postoperative results was 4.3 ± 5.9 mm (statistically insignificant), and the difference in COR position in the axial plane for preoperative vs. postoperative results was 1.6 ± 3.3 mm (statistically insignificant) (Table 3). For the inclination angle and anteversion angle, the differences between preoperative and postoperative results were 12.4° for the inclination angle (statistically significant; preoperative results 60.9 ± 8.5° and postoperative results 48.5 ± 5.5°) and 8.7° for the anteversion angle (statistically significant; preoperative results 24.9 ± 4.2° and postoperative results 16.3 ± 2.1°) (Table 3).

## 4. Discussion

The technological developments that have influenced orthopedics also include computer systems used in medicine. One such direction is the use of surgery planning software (3D Slicer 5.6.2) [12], which allows for very repeatable segmentation of bone structures, the assessment of the degree of damage to the pelvic bone defect, which we confirmed in our technical note (the difference in segmentation of bone structures of the undamaged side of the pelvis before and after the surgery was 0.01 ± 0.05% (Table 2)), the classification according to the classifications commonly accepted in orthopedics [6], the determination of the size of the acetabulum to be used in the revision surgery (Figure 3), and finally, postoperatively, the assessment of the positioning of the revision set elements and, thus, the prediction of the potential long-term effect of THA or RHA [1,6,11]. Unfortunately, as the number of THA procedures increases, the need for revision hip arthroplasty (RHA) also increases; it is estimated that an average 10–20% of THA patients will require RHA over the next 20 years [6,15]. The reasons for RHA are various, such as infection, osteolysis, aseptic loosening, and instability or dislocation [1,3,16]. A common consequence of the above-mentioned causes is significant loss of pelvic bone tissue, which includes, among others, defects in the anterior column of the acetabulum, which should be filled in an appropriate manner by trying to restore both the COR and the continuity of the pelvic BRIM, while maintaining the anteversion and inclination angles within the Lewinnek safe zone [17]. One of the techniques that we can use in such cases is the use of large-diameter cementless cups. As a rule, this type of cups allows for greater contact with the patient’s pelvic bone (it is assumed that the contact of the cup at the point of contact with the ischium, the pubic bone, and the roof is necessary), and thus obtains primary stability of the implant. However, in the literature it can be found that the use of jumbo cups may cause some difficulties in opening the COR, as it is assumed that this type of cup causes the COR to shift upward in the vertical position by an average of 10 mm [18]. Peng et al. [14] determined the COR shift relative to the normal/anatomical side of the pelvis to be an average of 7.6 ± 5.6 mm (upward) in the vertical position and 0.5 ± 5.1 mm in the horizontal position, while Shen et al. [19] determined the COR shift in large defects (AAOS III) to be 11.25 ± 5.1 mm, and in defects classified according to AAOS I to III, an average of 7.24 ± 3.85 mm. Similar results were obtained by Zhang et al. [13], i.e., a change of an average of 8.2 mm (upward). Comparing these data with our results, the difference between preoperative and postoperative results was 7.8 ± 9.3 mm in the vertical axial plane (coronal plane), while the difference in COR position in the horizontal axial plane (coronal plane) for preoperative vs. postoperative results was 4.3 ± 5.9 mm, and the difference in COR position in the axial plane for preoperative vs. postoperative results was 1.6 ± 3.3 mm (this parameter is not used in classical COR determination based on X-ray images). We can see that using VSP can significantly support the surgical team in properly opening the COR while simultaneously opening the pelvic BRIM (Figure 4). A similar situation occurs in the case of the inclination angle and anteversion angle, where the mean values of the inclination angle in the literature are 40.8 ± 5.1°, as obtained by Zhang et al. [13], and, in the case of Shen et al. [19], 44.40 ± 5.1°. The anteversion angle values are 14.9 ± 5.1° [13] and 16.85 ± 5.1° [19]. In our study, the mean value of the inclination angle was 60.9 ± 8.5°, and the mean value of the postoperative angle was 48.5 ± 5.5° The mean value of the anteversion angle in the preoperative assessment was 24.9 ± 4.2°, while the mean value of the postoperative inclination angle was 16.3 ± 2.1°. The obtained values fit perfectly within the Lewinnek safe zone. At the same time, we can see that VSP clearly supports surgeons in the correct positioning of the revision cup during the procedure, as exemplified by the above-mentioned values of the angles determined during VSP for pre- and postoperative analysis. Unfortunately, the jumbo cup technique has some disadvantages, such as the possibility of acetabular dislocation. Studies conducted by other teams showed a significant incidence of dislocation with the use of jumbo cups, ranging from 9.3% to 21.0% [13]. To prevent this phenomenon, we decided to use dual-mobility systems (DM) in our clinical practice, which have been used since 1970 and bring very good clinical results in revision surgeries as well [20]. Hartzler et al. [21] reported that the risk of dislocation was three times higher with conventional revision cups compared with dual-mobility cups, and the risk of re-revision due to dislocation was seven times higher with conventional revision cups. The data come from comparative retrospective studies assuming the use of a large 40 mm head diameter compared with the equivalent in the form of a dual-mobility set. The analysis period included a 3.5-year follow-up. Similar results were obtained by Chalmers et al. [22] using similar assumptions, i.e., a head diameter of 36 mm or greater, but with a shorter follow-up period of 2 years. Hence, a natural way to prevent such cases is to use dual-mobility systems, which we used in patients with very good results in the medium-term assessment, a follow-up of up to 12 months, where during the outpatient clinic visit information was collected as to whether a dislocation or aseptic loosening had occurred and whether the patient had experienced pain. The osseointegration was also assessed based on an X-ray examination.

A common consequence of aseptic loosening is the significant pelvic bone loss mentioned above, which cannot always be avoided without supplementation to ensure primary implant stabilization, even when using jumbo cups. In such cases, the use of allogenic transplants is a common practice. Allografts can be in morselized (chips) or structural (bulk) forms and can be used in cemented and non-cemented cups. In terms of the scope of our technical note, i.e., the Ghanem and Andreas type III classification of defects without pelvic discontinuity and the use of cementless dual-mobility cups, the literature data indicate common success estimates of 90% (95% CI 87–93), with the morselized allograft (91% (87–94)) showing a higher success rate than the bulk allograft by 3 percentage points (88% (81–93)), but the presented results were not statistically significant [23]. In relation to our technical note, it is difficult to analyze the long-term effect of the allograft application at this stage, but it should be stated that this was not the aim of our work. One very valuable observation resulting from our work is that VSP and 3D models allow determining the necessity of using allografts or augments, and possibly the way to position them. Another reason for the additional bone defect is the necessity of removing the primary acetabulum [15] and, sometimes, the inattentive use of cup reamers by the orthopedist. Another advantage of 3D models is that they allow for a very simple assessment of the geometric dimensions of the damaged areas of the pelvic bone and, on this basis, the selection of the appropriate diameter of the revision cup (Figure 2 and Figure 4) and the determination of the necessity of using the above-mentioned metallic augments, or possibly the use of an allograft (Figure 2 and Table 1) or other elements included in the revision sets (e.g., a cup–cage system). Unfortunately, our technical note has significant limitations, the first of which is the number of operated patients in relation to other works by other teams. This limitation is primarily due to the adopted patient classification (the same number of male and female patients, the same reason for the revision surgery and the extent of pelvic bone damage, limitation to the anterior column area, and the narrow dimensions of the dual-mobility revision cup and head used (a cup with dimensions from 62 mm to 68 mm and a head diameter of 28 mm). The second limitation is the observation for a period of no longer than 12 months. The basic observations conducted in this technical note concerned the very early postoperative period, i.e., 7 days after the procedure and during 12 months in the case of the first patients (the collected data were limited to a medical interview during a follow-up visit at the Orthopaedic Clinic). The first limitation, paradoxically, can be considered as an advantage due to the well-defined study group, which in the case of a planned long-term follow-up is necessary for the correct assessment of the procedures performed, which the authors are aware of and are currently working on.

## 5. Conclusions

Aseptic loosening and instability prove to be leading causes of revision failure. Solutions that involve supporting, planning, and carrying out surgery should be implemented as a preventive method for re-revision. Among them, virtual surgical planning and 3D anatomic models prove to be efficient in preoperative non-cemented RHA planning in patients with anterior column deficiency and pelvic BRIM discontinuity. This, in consequence, allows for obtaining primary stability of the revision cup and its osteointegration after surgery. Moreover, preservation of the COR and Lewinnek safe zones are crucial for prediction of good medium- and long-term revision assessment. The cementless dual-mobility system protects the prosthesis from dislocation in patients with a positive Trendelenburg sign and soft tissue envelope deficiency.

## Figures and Tables

**Table 1:** Clinical information on the planned and used acetabular sizes and decisions regarding the use of allograft vs. VSP.

Patient [Sex]	Acetabular Size According to VSP [mm]	Acetabular Size After Revision Surgery [mm]	Decision to Use Allograft According to VSP	Use of Allograft in Revision Surgery	Prosthesis Head [mm]	Screws
1 [male]	66	64	+	+	28	Two screws placed in acetabular roof
2 [male]	68	66	+	+		
3 [female]	64	66	+	+		
4 [male]	66	64	+	+		
5 [female]	62	64	+	+		
6 [male]	68	68	+	+		
7 [female]	64	62	+	+		
8 [male]	66	68	+	+		
mean ± SD	65.5 ± 2.1	65.3 ± 2.1				

**Table 2:** Comparison of the volume of segmented bone structures within the operated pelvis (mean ± SD, *n* = 8, *—statistically significant, *p* < 0.05).

Parameters	% Difference [Mean ± SD]	Range Min–Max [mm^3^]	Correlation Coefficient (Pearson r)	*p* Value
Determination of the correctness of pelvic bone segmentation—Comparison of the volume of the undamaged side of the pelvic bone before and after revision surgery (based on two CT scan results)	0.01 ± 0.005 *	180,150–360,838	Perfect line	0.0078
Determination of anterior column bone loss between the undamaged and the damaged pelvic bone—before revision surgery	25.2 ± 5.8 *	124,862–360,824	0.9904	0.0078
Determination of anterior column bone loss between the undamaged and the damaged pelvic bone—after revision surgery	30.1 ± 6.7 *	115,965–360,838	0.9857	0.0078
Determination of anterior column bone loss between damaged pelvic bones—before and after revision surgery	5.0 ± 2.0 *	124,862–284,985	0.9980	0.0078

**Table 3:** Differences in the COR position and differences in the inclination angle and anteversion angle values (mean ± SD, *n* = 8, *—statistically significant, #—statistically insignificant, *p* < 0.05), B column: AaPre-op: anteversion angle pre-operation values, AaPost-op: anteversion angle post-operation values, IaPre-op: inclination angle pre-operation values, IaPost-op: inclination angle post-operation values). Preoperative vs. postoperative results.

Parameters Differences for Operated Side of Pelvis	Difference [mm] (Mean ± SD)	Range Min–Max [mm]	Correlation Coefficient (Pearson r)	*p* Value	B [°]
Difference in COR position, vertical axial coronal plane	7.8 ± 9.3 *	22.2–49.2	0.4383	0.0493	-
Difference in COR position, horizontal axial coronal plane	4.3 ± 5.9 ^#^	24.5–42.7	0.5779	0.0787	-
Difference in COR position, axial plane	1.6 ± 3.3 ^#^	28.8–68.5	0.9659	0.2240	-
Difference in inclination angle	12.4° *	-	−0.3630	0.0025	IaPre-op: 60.9 ± 8.5 IaPost-op: 48.5 ± 5.5
Difference in anteversion angle	8.7° *	-	0.1324	0.0080	AaPre-op: 24.9 ± 4.2 AaPost-op: 16.3 ± 2.1

**Table 4:** Three-dimensional printing parameters of pelvic models [11].

3D Printing Parameters	
Printing temperature	205 °C
Build plate temperature	60 °C
Layer high	0.2 mm
Infill	60%
Support material	Polyvinyl alcohol (PVA) (Ultimaker B.V, Geldermalsen, The Netherlands)

## Data Availability

The original contributions presented in the study are included in the article, further inquiries can be directed to the corresponding author.
